# Threshold of Musculoskeletal Pain Intensity for Increased Risk of Long-Term Sickness Absence among Female Healthcare Workers in Eldercare

**DOI:** 10.1371/journal.pone.0041287

**Published:** 2012-07-20

**Authors:** Lars L. Andersen, Thomas Clausen, Hermann Burr, Andreas Holtermann

**Affiliations:** 1 National Research Centre for the Working Environment, Copenhagen Ø, Denmark; 2 Federal Institute for Occupational Safety and Health (BAuA), Berlin, Germany; The James Cook University Hospital, United Kingdom

## Abstract

**Purpose:**

Musculoskeletal disorders increase the risk for absenteeism and work disability. However, the threshold when musculoskeletal pain intensity significantly increases the risk of sickness absence among different occupations is unknown. This study estimates the risk for long-term sickness absence (LTSA) from different pain intensities in the low back, neck/shoulder and knees among female healthcare workers in eldercare.

**Methods:**

Prospective cohort study among 8,732 Danish female healthcare workers responding to a questionnaire in 2004–2005, and subsequently followed for one year in a national register of social transfer payments (DREAM). Using Cox regression hazard ratio (HR) analysis we modeled risk estimates of pain intensities on a scale from 0–9 (reference 0, where 0 is no pain and 9 is worst imaginable pain) in the low back, neck/shoulders and knees during the last three months for onset of LTSA (receiving sickness absence compensation for at least eight consecutive weeks) during one-year follow-up.

**Results:**

During follow-up, the 12-month prevalence of LTSA was 6.3%. With adjustment for age, BMI, smoking and leisure physical activity, the thresholds of pain intensities significantly increasing risk of LTSA for the low back (HR 1.44 [95%CI 1.07–1.93]), neck/shoulders (HR 1.47 [95%CI 1.10–1.96]) and knees (HR 1.43 [95%CI 1.06–1.93]) were 5, 4 and 3 (scale 0–9), respectively, referencing pain intensity of 0.

**Conclusion:**

The threshold of pain intensity significantly increasing the risk for LTSA among female healthcare workers varies across body regions, with knee pain having the lowest threshold. This knowledge may be used in the prevention of LTSA among health care workers.

## Introduction

Sickness absence from work is considered a global health indicator [1]. Long-term sickness absence (LTSA) is especially relevant, because workers being absent for several consecutive weeks have increased risk for not returning to the labor market [2]. Because being gainfully employed plays an important role in well-being and societal identity [3] prevention of LTSA should have high priority. Knowledge of prognostic factors for LTSA is important for optimally targeting preventive efforts.

More than 100 million European citizens suffer from chronic musculoskeletal pain [4], and musculoskeletal disorders are the most common causes of work disability and consequent absence from work [5]. Low back pain and neck/shoulder pain are associated with both short-term sickness absence and LTSA in several occupations [6]–[11]. However, the consequence of pain from different body regions and severities of pain may vary between occupations with different physical demands. For example, whereas knee pain did not predict LTSA in the general working population [12], knee pain was a strong risk factor for LTSA among healthcare workers in eldercare [13]. However, previous studies have used different definitions and cut-points of pain severity making comparisons between the types of pain and occupations difficult. Thus, the association between musculoskeletal pain and risk of sickness absence should be evaluated separately for each specific occupation, body part and thresholds of pain intensity.

The prevalence of musculoskeletal disorders and LTSA is high in occupations with physically demanding work [14]. Healthcare work is particularly physically demanding [15], and in a survey involving more than 8000 healthcare workers in eldercare 23%, 28%, and 12% reported chronic pain (>30 days last year) in the low back, neck/shoulders, and knees, respectively [13]. Thus, healthcare work in eldercare may be particularly physically demanding. In spite of preventive efforts in the healthcare sector – e.g. provision of manual handling equipment – elimination of all incidences of musculoskeletal pain is probably unrealistic. Thus, prevention of the consequences of musculoskeletal pain may be more realistic. To provide better recommendations for protecting individual healthcare workers from LTSA and consequent job loss there is a need for knowing when pain intensity reaches a critical level for increasing the risk of LTSA.

The aim of our study was to estimate the risk for long-term sickness absence (LTSA) from different pain intensities in the low back, neck/shoulder and knees among female healthcare workers in eldercare.

## Methods

### Population

The present study is based on survey data collected among employees in the eldercare-services merged with the Danish Register for Evaluation of Marginalization (DREAM), which is a National register on social transfer payments [16]. A survey on health and working conditions among 12,744 employees in the eldercare-sector of 36 Danish municipalities was conducted in 2004–5, yielding a response percentage of 78% (9,947 persons). All respondents of the survey were identified and followed in the DREAM register for one year after completion of the survey. Employees being engaged in management or in production of services not directly related to the provision of health-related care services (e.g. kitchen staff, janitors, administrators) were excluded from the analyses (N = 995). Further, male respondents were excluded (N = 220). Thus, the target population comprised 8,732 female employees being directly engaged in the provision of health-related care services in the Danish eldercare-sector (e.g. nurses, nurses aides).

### Ethical approval

The study has been notified to and registered by Datatilsynet (the Danish Data Protection Agency). According to Danish law, questionnaire and register based studies do not need approval by ethical and scientific committees, nor informed consent [17], [18]. All data was de-identified and analyzed anonymously.

### Outcome variable: Long-term sickness absence

Data on sickness absence were drawn from the DREAM register [16], [19], and linked to the survey data via the unique personal identification number given to all Danish citizens at birth. The DREAM register contains weekly information on granted sickness absence, education, employment, disability pension etc for all citizens in Denmark. Due to Danish law the reason for sickness absence is not registered. Sickness absence compensation is given to the employer, who can apply for a refund from the state for employees after two weeks of sickness absence. Because the employer has a strong economic incentive to report sickness absence, the validity of the sickness absence data has been found to be high [16]. Long-term sickness absence was defined as the occurrence of a period of eight or more consecutive weeks of sickness absence in a one-year follow-up period from the date of the questionnaire reply. We chose an absence period for eight or more consecutive weeks as empirical evidence indicates that employees who are absent for eight weeks or more have a substantially increased risk for not returning to work [2].

### Risk factor: Intensity of musculoskeletal pain

Participants rated pain in the low back, neck/shoulders, and knees, respectively, as average pain during the last three months on a numerical rating scale from 0–9, where 0 is ‘no pain’ and 9 is ‘worst imaginable pain’. The rating scale was horizontally oriented to represent a modified visual-analogue scale [20]. A drawing from the Nordic Questionnaire defined the three respective body regions [21]. Respondents with pain intensities of 0 were set as reference.

To ease the discussion we term pain intensities of 0–2 as ‘low’, 3–5 as ‘moderate’ and more than 5 as ‘severe’.

### Confounders

Potential confounders included age, body mass index (BMI = weight/height^2^), smoking status (dichotomous variable depicting current smoker/non-smoker), seniority (years working as healthcare worker; continuous variable), leisure physical activity (4-categories from low to a very high level of leisure physical activity) [13], [22], physical workload based on the Hollmann Index (scale of 0–62, with 62 representing the highest degree of physical workload) questionnaire asking about body postures and weight lifted during the working day [23], and four indicators of psychosocial work conditions from the Copenhagen Psychosocial Questionnaire (COPSOQ) [24], [25]: emotional demands, role conflicts, influence at work, and quality of leadership (normalized on a 0–100 scale according to the test-score manual).

### Statistics

Using the Cox proportional hazards model, we estimated the risk of pain intensities from 1 to 9 for onset of LTSA, referencing pain intensity of 0. Hazard ratios (HR) and 95% confidence intervals (95% CI) were calculated separately for the three body regions. Smoking status and leisure time physical activity were treated as categorical variables in the analysis. Age, BMI, tenure, physical workload and the four indicators of psychosocial work conditions were treated as continuous variables. Respondents were followed in the DREAM-register for one year and respondents were censored after first case of LTSA. Respondents were furthermore censored in case of retirement, immigration or death. In Model 1 we adjusted for age. In Model 2 we additionally adjusted for life-style related factor (BMI, leisure physical activity and smoking status). In Model 3 we additionally adjusted for work-related factors (seniority, physical workload, and psychosocial work conditions). The data on LTSA correspond to survival times which in most cases are censored as the cohort is only followed for one year. When individuals had an onset of LTSA in the one-year follow-up period, the survival times were non-censored and referred to as event times. The estimation method was maximum likelihood and the PHREG procedure of SAS 9.2 was used. We included the TEST statement in the PHREG procedure to test the proportional hazards assumption. We used the LIFETEST procedure of SAS 9.2. to produce Kaplan-Meier curves for a visual representation of the hazards.

## Results

Table 1 presents descriptive data for the main study variables. Of the 8,732 female healthcare workers 38%, 37% and 18% had moderate pain and 12%, 17% and 6% had severe pain in the low back, neck/shoulders and knees, respectively. Only 0.5% had severe pain in all three regions. Moreover, 6.3% of the respondents developed at least one period of LTSA during the follow-up year. In comparison, among non-respondent females 11.0% had at least one period of LTSA during the survey period or follow-up year.

**Table 1:** Descriptive statistics for the main study variables.

	Mean (SD) or pecentage
Long-term sickness absence (%)	6.3%
Age (years)	45 (10)
**Life-style related factors**	
Body Mass Index (kg·m^−2^)	25 (4)
Smoker (%)	37%
Leisure physical activity (%)	
Low	5%
Medium	42%
High	49%
Very high	5%
**Work related factors**	
Seniority (years)	9 (7)
Physical workload (Hollmann Index, scale 0–62)*	20 (10)
Psychosocial working conditions (0–100)§	
Emotional demands	46 (19)
Influence at work	45 (21)
Role conflicts	42 (16)
Quality of leadership	57 (22)
**Musculoskeletal pain**	
Low back pain (%)	
Pain intensity 0–2	50%
Pain intensity 3–5	38%
Pain intensity >5	12%
Neck/shoulder pain (%)	
Pain intensity 0–2	46%
Pain intensity 3–5	37%
Pain intensity >5	17%
Knee pain (%)	
Pain intensity 0–2	76%
Pain intensity 3–5	18%
Pain intensity >5	6%

Values are given as means (SD) or percentage of the female healthcare workers (N = 8,732).

* ) Higher values indicate higher physical workloads.

§ ) Higher values indicate higher levels of Emotional demands, Role conflicts, Influence at work and Quality of leadership.

**Figure 1:**
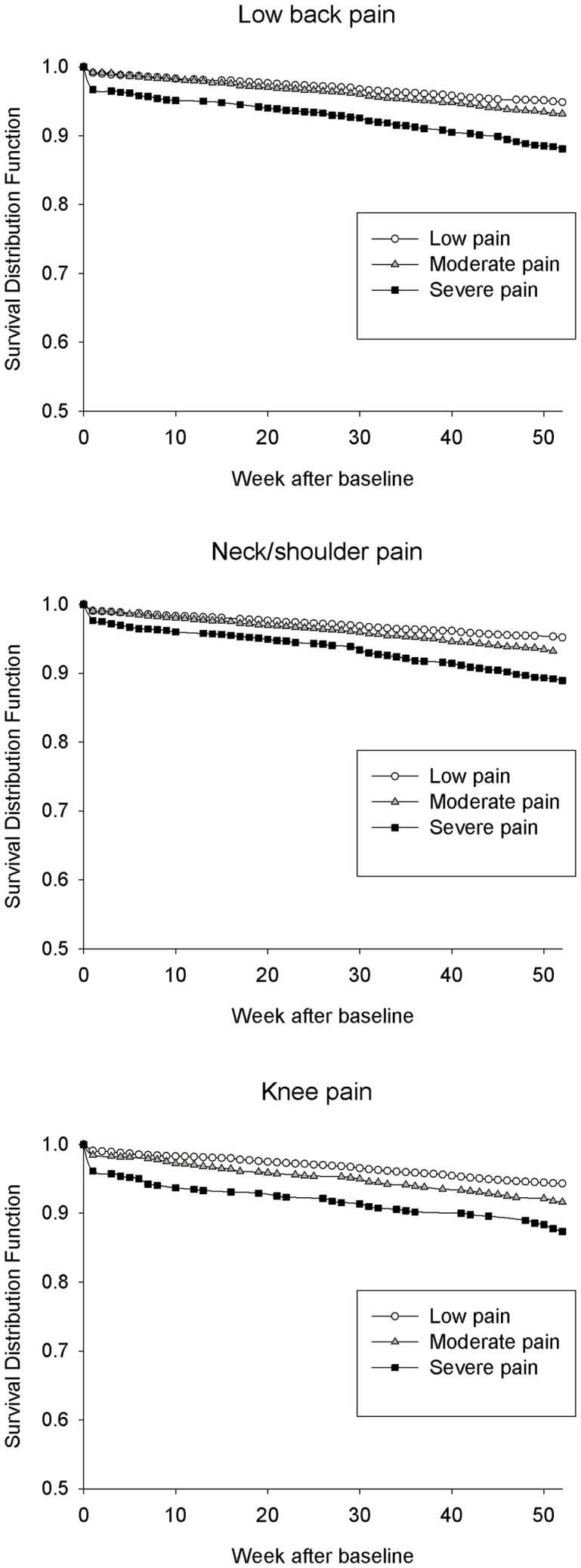
Visual representation of the hazards (Kaplan-Meier curves) at 0–52 weeks from baseline for low back pain, neck/shoulder pain and knee pain, respectively. The Y-axis represents the proportion of female healthcare workers not having LTSA. Pain intensity is stratified into low (0–2), medium (3–5) and severe (>5) pain on a scale of 0–10.

Figure 1 shows a visual representation of the hazards. Table 2 summarizes pain intensities from 1 to 9 (reference: 0) in the different body regions for the risk of LTSA. Trend tests for the relationship between increasing pain intensities and increasing risk of LTSA was highly significant for all three body regions (P<0.001) (not shown in Table 2). With adjustment for age (Model 1), the threshold of pain intensities for significantly increased risk of LTSA was 5, 4 and 3 (scale 0–9) for the low back, neck/shoulders and knees, respectively. With additional adjustment for life-style related factors (Model 2) these findings remained. At the upper boundary of the scale, pain intensities of 8–9 for the different body regions resulted in three- to fivefold increased risk for LTSA. With additional adjustment for work-related factors (Model 3) the hazard ratios generally decreased and the thresholds for significantly increased risk of LTSA was 7, 7 and 5 (scale 0–9) for the low back, neck/shoulders and knees, respectively.

**Table 2:** Hazard ratios (HR) and 95% confidence intervals for onset of long-term sickness absence during 12 months follow-up for the different levels of pain intensity (scale 0–9) for the low back, neck/shoulders, and knees.

		Model 1		Model 2		Model 3	
	n	HR	95% CI	HR	95% CI	HR	95% CI
Low back pain (scale 0–9)							
0	2811	1	-	1	-	1	-
1	472	0.64	(0.38–1.07)	0.59	(0.34–1.02)	0.53	(0.29–0.95)
2	949	0.95	(0.69–1.32)	0.93	(0.67–1.31)	0.87	(0.61–1.23)
3	1364	1.16	(0.88–1.52)	1.11	(0.84–1.46)	0.99	(0.74–1.33)
4	1047	1.19	(0.89–1.59)	1.14	(0.85–1.53)	0.97	(0.71–1.33)
5	851	1.51	(1.13–2.02)	1.44	(1.07–1.93)	1.26	(0.93–1.72)
6	532	1.55	(1.11–2.18)	1.43	(1.00–2.04)	1.19	(0.82–1.72)
7	339	2.46	(1.75–3.47)	2.37	(1.67–3.35)	2.03	(1.41–2.92)
8	86	5.23	(3.31–8.27)	4.97	(3.10–7.98)	4.17	(2.55–6.84)
9	74	3.96	(2.20–7.13)	4.28	(2.37–7.74)	3.43	(1.79–6.57)
Neck/shoulder pain (scale 0–9)							
0	2700	1	-	1	-	1	-
1	375	0.86	(0.51–1.45)	0.83	(0.48–1.44)	0.83	(0.47–1.47)
2	869	0.83	(0.58–1.21)	0.90	(0.62–1.30)	0.91	(0.63–1.34)
3	1156	1.15	(0.86–1.55)	1.17	(0.86–1.58)	1.18	(0.86–1.62)
4	1042	1.50	(1.14–1.99)	1.47	(1.10–1.96)	1.32	(0.97–1.79)
5	946	1.38	(1.03–1.87)	1.42	(1.04–1.92)	1.26	(0.91–1.74)
6	652	1.55	(1.12–2.15)	1.54	(1.10–2.16)	1.21	(0.84–1.75)
7	522	2.28	(1.67–3.12)	2.25	(1.64–3.10)	1.95	(1.39–2.72)
8	218	3.58	(2.46–5.19)	3.44	(2.35–5.03)	2.74	(1.82–4.13)
9	98	4.03	(2.43–6.68)	4.25	(2.52–7.15)	3.86	(2.27–6.56)
Knee pain (scale 0–9)							
0	5637	1	-	1	-	1	-
1	296	1.17	(0.74–1.86)	1.30	(0.82–2.08)	1.42	(0.89–2.26)
2	584	0.97	(0.67–1.40)	0.92	(0.63–1.36)	0.86	(0.57–1.29)
3	689	1.39	(1.03–1.87)	1.43	(1.06–1.93)	1.32	(0.96–1.81)
4	456	1.52	(1.08–2.15)	1.44	(1.01–2.06)	1.39	(0.96–2.01)
5	354	1.82	(1.27–2.61)	1.85	(1.28–2.67)	1.72	(1.17–2.51)
6	224	1.35	(0.82–2.23)	1.18	(0.69–2.02)	1.16	(0.67–1.99)
7	172	3.16	(2.13–4.70)	3.16	(2.12–4.70)	3.22	(2.14–4.84)
8	93	2.93	(1.68–5.10)	2.99	(1.71–5.21)	2.84	(1.62–4.97)
9	54	3.77	(2.00–7.09)	3.27	(1.61–6.63)	2.90	(1.42–5.96)

Model 1: Adjusted for age.

Model 2: Adjusted for age, BMI, smoking, and leisure physical activity.

Model 3: Adjusted for age, BMI, smoking, leisure physical activity, seniority, physical workload, and psychosocial work environment.

## Discussion

Our study showed that thresholds of pain intensity increasing the risk for LTSA vary across body regions, with knee pain having the lowest threshold. With adjustment for life-style related factors the findings remained, but the hazard ratios decreased when adjusting for work-related factors.

In our study, moderate to severe pain from the low back, neck/shoulder and knees were significant risk factors for LTSA among healthcare workers. Importantly, specific thresholds for each body region existed, with pain intensity thresholds of 5, 4 and 3 for the low back, neck/shoulders and knees, respectively, referencing pain intensity of 0 (Model 2). Prospective cohort studies have documented that pain from the back, neck and shoulders among different occupational groups increase the risk for sickness absence by a range from 30% to 390% [6]–[11], [26]. Differences in definitions and specific cut-points of pain severity between the studies as well as inclusion of different occupational groups may explain this wide range in risk estimates. Our study elaborates on these previous findings by documenting specific thresholds of pain intensity for significantly increased risk of long-term sickness absence in female healthcare workers.

An unexpected finding is the relatively high threshold for low back pain, i.e. 5 on a scale of 0–9, compared with the thresholds of 3–4 for the other body regions. Even with minimal adjustment for other factors associated with sickness absence (Model 1) the HR's for pain intensities below 5 was close to 1. As a possible explanation, the healthcare sector has during the last decades introduced several initiatives to manage work in spite of low back pain – for example back schools and provision of manual handling equipment. Also, many countries have provided much public information about the benefits of staying active in spite of back pain [27].

The pain intensity threshold of 4 in the neck/shoulders for increased risk of LTSA among the healthcare workers in our study is roughly in line with a previous study in sewing machine operators showing that clinical findings occurred more frequently with moderate levels of self-reported complaints [28]. In that study, a summation of four complaint scores on a scale of 0–9 (i.e. range 0–36), showed a cut-point of 12 (i.e. ∼3 on a scale of 0–9) for increased prevalence of myofascial pain syndrome and rotator cuff tendinitis. Further, a Danish study among the general working population showed that higher pain intensity in the neck/shoulder was related to increased risk of LTSA [29].

Knee pain intensities at 3 or above were associated with significantly increased risk for LTSA. Thus, although knee pain is less prevalent than low back and neck/shoulder pain, the consequences of individual knee pain appear to be higher among female healthcare workers. By contrast, among 5000 Danish employees from different occupations chronic knee pain, defined as at least 30 days with knee pain during the last year, was not a significant risk factor for LTSA [12]. Thus, the consequences of musculoskeletal pain in different body regions may vary across occupations and with different cut-points and definitions of pain. For example, employees in sedentary occupations may not experience the same consequences of knee pain as employees with strenuous physical labor. This stresses the importance of determining occupation-specific thresholds of pain intensity for increased risk of LTSA.

The hazard ratios decreased when adjusting for work-related factors, resulting in higher thresholds of pain intensity for increased risk of LTSA (Model 3). With adjustment for seniority, physical workload and psychosocial work conditions the thresholds for LTSA were 7, 7 and 5 for pain intensities in the low back, neck/shoulder and knees. We adjusted for these factors because physical as well as psychosocial working conditions are shown to be related to both the predictor (musculoskeletal pain) and the outcome (LTSA) [30]–[34]. In this regard, a good working environment may be viewed as a potential resource protecting workers from LTSA in spite of relatively high intensities of musculoskeletal pain. By contrast, if musculoskeletal pain simply mediates the relation between work exposures and LTSA then adjusting for these work related factors is not meaningful. Thus, asking only a single-item question about pain intensity, the thresholds of 5, 4 and 3 determined from Model 1 and 2 are likely more relevant for guidelines aiming to prevent the consequences of musculoskeletal pain.

Our study has both strengths and limitations. The large sample size of female healthcare workers from several different municipalities strengthens the validity of the estimates for this specific occupational group. However, the sample size of the reference groups as well as the sample size of each pain-intensity group also influences the range of the confidence intervals. Thus, statistically significant thresholds may have been found at lower pain intensities had the sample size been larger. Therefore, the practical relevance of our findings should also be considered. The 43% to 47% increased risk for LTSA from pain intensities of 5, 4, and 3 in the low back, neck/shoulder and knees (Model 2), respectively, seems highly relevant. By contrast, one level below the statistically significant thresholds the hazard ratios were near 1 ranging from 0.92 to 1.17 and may therefore not be relevant even if statistically significant with a larger sample size. Thus, the practical relevant thresholds are likely very near the statistically significant thresholds of 5, 4 and 3 determined in the present study. Due to the rather homogeneous group of female healthcare workers we did not control for socioeconomic factors. The inclusion criteria limit the generalizability of our findings to female healthcare workers in eldercare. As another limitation, recall bias regarding a three-month recall for musculoskeletal pain may exist. Also, due to the study design no causal relations can be established. Further, among the total target population of female healthcare workers 11.0% and 6.3% of the non-respondents and respondents, respectively, had LTSA during follow-up. Thus, response bias may exist, i.e. non-respondents may have poorer health than respondents. Because the present questionnaire survey was conducted at the workplace, employees on sick leave during the survey period did not have the opportunity to reply. Future studies should consider mailing questionnaire surveys to employees on sick leave during the study period. Furthermore, we had no information regarding co-morbitidies, e.g. osteoarthritis or fibromyalgia, which may also influence the thresholds, and could be a target for future research.

In conclusion, the threshold of pain intensity increasing the risk for LTSA among female healthcare workers varies across body regions, being 5, 4 and 3 (scale 0–9) for the low back, neck/shoulders and knees, respectively. This knowledge may be used to better protect individual healthcare workers from LTSA by initiating preventive actions when reporting pain intensities at or above the respective thresholds.
